# Gene Editing Therapies Targeting Lipid Metabolism for Cardiovascular Disease: Tools, Delivery Strategies, and Clinical Progress

**DOI:** 10.3390/cells15020134

**Published:** 2026-01-12

**Authors:** Zhuoying Ren, Jun Zhou, Dongshan Yang, Yanhong Guo, Jifeng Zhang, Jie Xu, Y Eugene Chen

**Affiliations:** Center for Advanced Models for Translational Sciences and Therapeutics, University of Michigan Medical School, 2800 Plymouth Road, Ann Arbor, MI 48109, USA; zhuoyren@umich.edu (Z.R.);

**Keywords:** gene editing therapy, cardiovascular diseases, lipid metabolism genes, clinical trials

## Abstract

**Highlights:**

**What are the main findings?**

- Gene editing therapies targeting liver-specific genes that are involved in lipid metabolism for treating cardiovascular diseases (LivGETx-CVD) have progressed from conceptual frameworks to early-phase clinical trials, with the potential to redefine prevention and long-term management of dyslipidemia and atherosclerotic cardiovascular diseases.
- Human genetics and preclinical studies suggest several promising genes, such as *PCSK9*, *ANGPTL3*, *IDOL*, *ASGR1*, *APOC3*, and *LPA*, for targeting in LivGETx-CVD to achieve durable LDL-C and triglyceride lowering after a single administration.

**What is the implication of the main findings?**

- LivGETx-CVD is a potential “vaccine-like” intervention for cardiovascular disease, offering long-term protection without the adherence challenges and access barriers of chronic lipid-lowering medications.
- To translate LivGETx-CVD beyond rare, severe dyslipidemias toward broader preventive use, the field must address outstanding challenges in delivery safety, off-target effects, cost-effectiveness, as well as ethical and regulatory oversight, requiring larger and longer clinical trials and societal-level discussion.

**Abstract:**

Gene editing technologies have revolutionized therapeutic development, offering potentially curative and preventative strategies for cardiovascular disease (CVD), which remains a leading global cause of morbidity and mortality. This review provides an introduction to the state-of-the-art gene editing tools—including ZFNs, TALENs, CRISPR/Cas9 systems, base editors, and prime editors—and evaluates their application in lipid metabolic pathways central to CVD pathogenesis. Emphasis is placed on targets such as *PCSK9*, *ANGPTL3*, *CETP*, *APOC3*, *ASGR1*, *LPA*, and *IDOL*, supported by findings from human genetics, preclinical models, and recent first-in-human trials. Emerging delivery vehicles (AAVs, LNPs, lentivirus, virus-like particles) and their translational implications are discussed. The review highlights ongoing clinical trials employing liver-targeted in vivo editing modalities (LivGETx-CVD) and provides insights into challenges in delivery, off-target effects, genotoxicity, and immunogenicity. Collectively, this review captures the rapid progress of LivGETx-CVD from conceptual innovation to clinical application, and positions gene editing as a transformative, single-dose strategy with the potential to redefine prevention and long-term management of dyslipidemia and atherosclerotic cardiovascular disease.

## 1. Introduction

The past decade has witnessed rapid advances in gene editing technologies. In 2020, the Nobel Prize in Chemistry was awarded to Drs. Charpentier and Doudna for their contributions in the development of CRISPR/Cas9 (Clustered Regularly Interspaced Short Palindromic Repeats/CRISPR-associated protein-9), the dominant gene editing nuclease (GENs) that is used nowadays [1]. Today, gene editing is widely used in research and is being explored in therapeutics. Gene editing therapy, referred to as GETx in this article, offers new options for treating and maybe curing many diseases, including genetic diseases, infectious diseases, and cancer.

Cardiovascular disease (CVD) remains one of the most significant health challenges worldwide, accounting for one-third of all global deaths and resulting in more than 17 million fatalities annually [2]. Elevated levels of circulating low-density lipoprotein cholesterol (LDL-C) have been identified as a major risk factor for CVD, as established by the Framingham Heart Study, a landmark longitudinal investigation into cardiovascular risk factors [3]. Similarly, elevated triglyceride (TG) levels are positively associated with an increased risk of atherosclerosis and cardiovascular diseases [4]. While current first-line therapies, such as statins, and emerging medicines, such as PCSK9 (proprotein convertase subtilisin/kexin type 9) monoclonal antibodies, are effective in slowing the progression of coronary artery disease in many [5,6,7], a considerable subset of patients do not benefit, due to reasons such as drug intolerance, fear of side effects, genetic factors affecting drug response, and non-adherence driven by prior medication experiences or aversion to drugs. As a result, there is a critical unmet need for innovative cardiovascular treatments that can more effectively lower disease risk while minimizing the burden of long-term medication adherence. New technologies such as gene editing, as we will see soon, are paving the way toward solutions that require fewer doses, offering hope for more sustained protection and improved patient quality of life.

**Figure 1:**
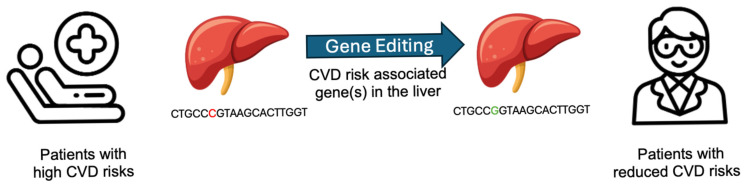
Illustration of LivGETx-CVD. Gene editing on CVD risk-associated gene(s) in the liver is a “one-shot, one-cure” therapy. A single administration is expected to provide long-term therapeutic protection.

Gene editing tools, represented by CRISPR/Cas9, hold promise for delivering new therapeutic solutions for many human diseases. The question whether GETx can be employed for treating cardiovascular diseases (GETx-CVD) was raised in 2014, one year after Cas9 debuted [8]. CVD, like many other diseases, is genetically predisposed; hence, modifying the gene(s) “for the good” may bring therapeutic benefits [9,10]. The approach is conceptually simple: (i) first, identify a gene whose loss-of-function mutation provides cardioprotective effects; these genes are often involved in lipid metabolism [9]. (ii) Next, employ gene editing technologies such as CRISPR/Cas9, or its advanced iterations like base or prime editors or other editing tools, to introduce these beneficial mutations. (iii) Such intervention confers a lasting reduction in cardiovascular disease risks. Analogous to vaccination, a single administration ideally provides long-term protection, leading some to refer to such a strategy as a form of “CVD vaccination” [11], as illustrated in Figure 1.

In this review, we first introduce the major gene editing tools and recent advances in in vivo delivery platforms. We then highlight key genetic targets for GETx-CVD. One of the best-characterized domains in cardiovascular genetics is how genes that regulate cholesterol and triglyceride metabolism and homeostasis drive atherosclerosis and downstream cardiovascular diseases. Principal players include low-density lipoprotein receptor (*LDLR*), proprotein convertase subtilisin/kexin type 9 (*PCSK9*), apolipoprotein B (*APOB*), inducible degrader of LDLR (*IDOL*), asialoglycoprotein receptor 1 (*ASGR1*), angiopoietin-like 3 (*ANGPTL3*), apolipoprotein C3 (*APOC3*), and lipoprotein(a) (*LPA*), which are all liver-specific or liver-relevant. We refer to GETx-CVD strategies directed at these hepatic lipid-metabolism genes as LivGETx-CVD and provide a focused review of their biological rationale and therapeutic potential. Finally, we summarize ongoing LivGETx-CVD clinical trials targeting these pathways.

## 2. Gene Editing Tools

**Figure 2:**
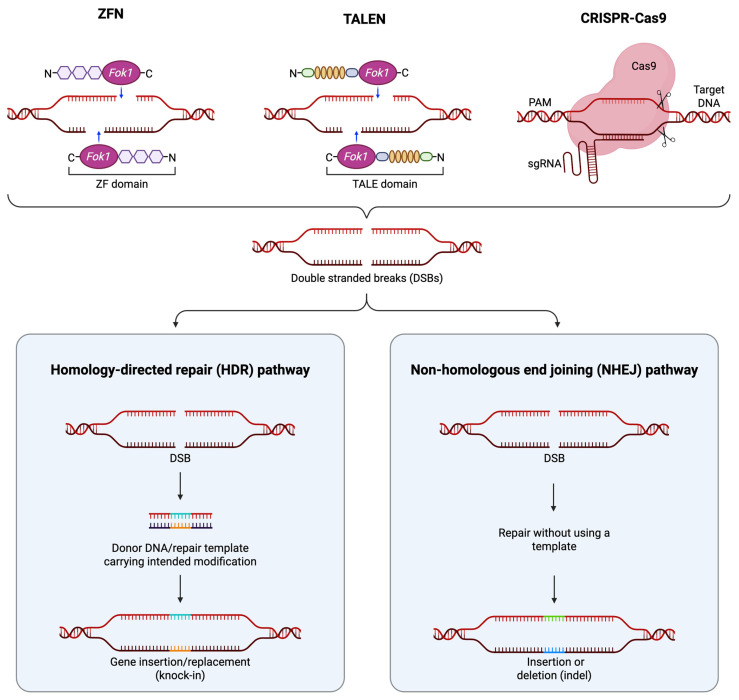
Illustration of ZFN, TALEN, and CRISPR-Cas9 and their shared downstream DNA repair pathways: HDR and NHEJ *. ZFN, TALEN, and CRISPR-Cas9 are all capable of generating DNA double-stranded breaks (DSBs), which are repaired by two major pathways: the homology-directed repair (HDR) utilizing endogenous or user-supplied donor template for precise repair; and the non-homologous end joining (NHEJ), which is error-prone and introduces insertion and deletion (indel) edits. * Modified from references [15,16] and created in BioRender. Xu, J. (2025) https://BioRender.com/m6qkiei (accessed on 1 December 2025).

Gene editing nucleases (GENs), represented by Zinc Finger Nuclease (ZFN), Transcription Activator-Like Effector Nuclease (TALEN), and CRISPR/Cas9, have become major tools in biomedical research [12]. ZFN, TALEN, and CRISPR/Cas9 are all capable of generating double-strand breaks (DSB) at specific genomic loci in a programmable (so-called customizable) manner (Figure 2). These tools opened the era of gene editing. In recent years, base editors (BEs) and PRIME editors (PEs) have emerged as prominent tools in both research and clinical applications. Here we provide a brief introduction to these tools. Comprehensive reviews of gene editing tools, including other recent developments, are available elsewhere [13,14].

### 2.1. Zinc-Finger Nucleases

ZFN is the first generation of GEN, which became commercially available in the early 2000s. A ZFN pair comprises two different zinc-finger proteins (DNA-binding domains), each linked with a Fok I endonuclease domain [17]. In the most common practice, a zinc finger domain would consist of 6 tandem fingers, each recognizing 3 bp of DNA, to recognize an 18 bp-long sequence. The two zinc-finger domains lie in inverted orientation, such that the two FokI endonuclease domains would form a functional dimer to cleave the target DNA sequence.

### 2.2. Transcription Activator-like Effector Nucleases

The TALEN is considered an upgraded version of programmable nucleases. Like ZFN, TALEN recognizes targeted DNA sequence through programmable protein domains, so-called transcription activator-like effectors (TALEs), which were derived from *Xanthomonas* spp. bacteria [18,19], and also uses the FokI pair as DNA scissors. Each TALE contains a repeated highly conserved 33–34-amino-acid sequence with Repeat Variable Diresidue (RVD) at the 12th and 13th amino acid positions. The RVDs recognize specific nucleotides, for example, NN for guanine, NI for adenine, HD for cytosine, and NG for thymine, etc. This straightforward relationship between amino acid sequence and DNA recognition in TALEN design has allowed for easier engineering of sequence-specific binding domains than with ZFNs [15,16].

### 2.3. CRISPR/Cas9

The CRISPR/Cas9 technology is the most recent version of the programmable genome editing nucleases. In its most popular format, the CRISPR/Cas9 ribonuclease complex consists of a Streptococcus pyogenes Cas9 (SpCas9) protein and a single guide RNA (sgRNA). SpCas9 recognizes and binds to a protospacer adjacent motif (PAM) sequence on the target DNA, initiating local DNA unwinding that allows the guide RNA to form a complementary RNA–DNA hybrid (R-loop) with the target sequence; upon complete base pairing, conformational changes activate the HNH and RuvC nuclease domains of SpCas9, resulting in site-specific cleavage of both DNA strands three base pairs upstream of the PAM. One distinction of CRISPR/Cas9 from ZFN and TALEN is that CRISPR/Cas9 recognizes the target sequence through RNA–DNA complementation. This feature makes CRISPR/Cas9 much easier and faster to design and engineer than the two prior versions, which rely on protein–DNA recognition.

### 2.4. Nuclease-Inactive Cas9 Variants

ZFN, TALEN, and CRISPR/Cas9 are capable of generating DSBs. The DSBs are subsequently repaired by different pathways, with non-homologous end joining (NHEJ) as the dominant one, along with others such as homology-directed repair (HDR) and alternative NHEJ (alt-NHEJ), leading to different editing outcomes [20]. As its name suggests, NHEJ is imprecise and leads to undesired insertion and deletion (indel) events at the target site as well as the off-target sites throughout the genome. One strategy to mitigate the potential adverse effects of NHEJ is to prevent double-strand break (DSB) formation by using a nuclease-inactive Cas9 variant generated through mutating the HNH and/or the RuvC domains. The resulting deactivated Cas9 (dCas9) lacks endonuclease activity and does not generate DSBs at all, whereas the nickase Cas9 (nCas9) variant cleaves only a single strand of the DNA.

### 2.5. Base Editors

**Figure 3:**
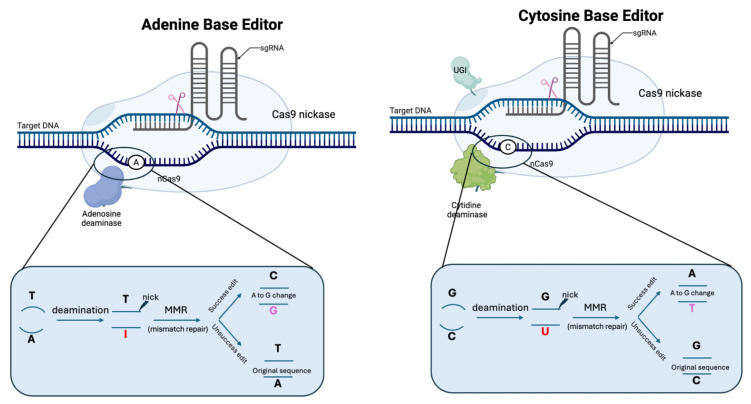
Illustration of adenine base editor (ABE) and cytosine base editor (CBE) *. Base editors use Cas9 nickase (nCas9) to introduce single-stranded breaks at the target strand, followed by the deaminase that is often fused to the N-terminus of Cas9 to edit the “A” (in ABE) or “C” (in CBE) that is within the editing window on the non-targeted strand. Base editors exploit the mismatch repair (MMR) mechanism to achieve the desired base editing outcome. UGI: Uracil Glycosylase Inhibitor. * Created in BioRender. Xu, J. (2025) https://BioRender.com/pkbwuq4 (ABE, accessed on 1 December 2025), and Xu, J. (2025) https://BioRender.com/eosz6kw (CBE, accessed on 1 December 2025).

Base editors were developed by fusing deaminases, such as cytidine deaminase APOBEC or adenine deaminase engineered TadA, with nuclease-inactive Cas9 [21,22,23,24]. The base editor ribonuclease complex is located at the target sequence with the help of the guide RNA, followed by the deaminase working to deaminate bases within the editing window. A cytidine base editor (CBE) converts C:G to T:A; whereas an adenine base editor (ABE) converts A:T to G:C. Other types of base editors capable of other types of base conversions and transversions have also been developed in recent years [25,26,27]. Compared with the DSB-based editing method, the base editors provide a powerful route to efficiently install or correct point mutations with much less genotoxicity concern. Figure 3 illustrates the two major types of BEs: CBE and ABE.

### 2.6. PRIME Editors

**Figure 4:**
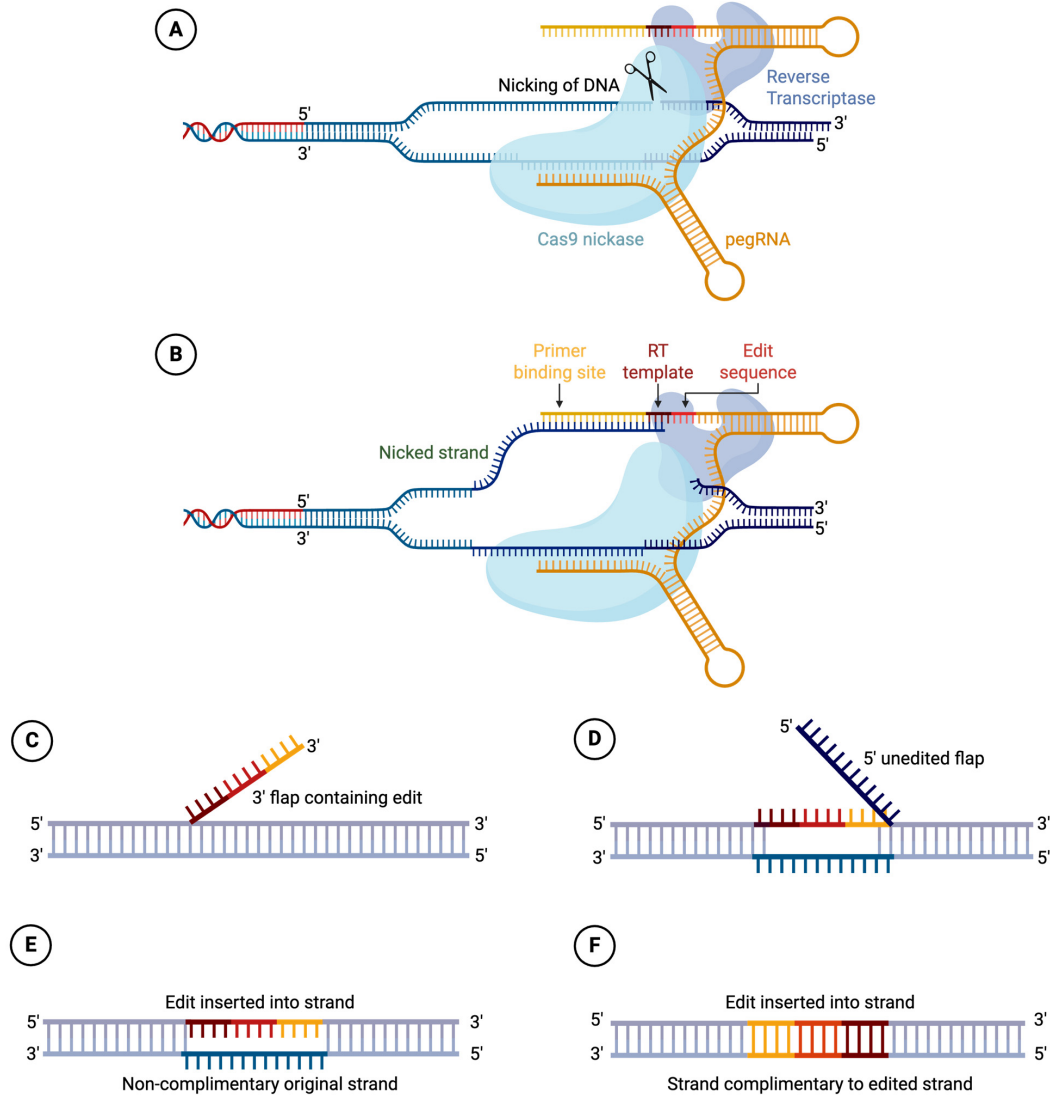
Illustration of PRIME editor *. Prime editor’s protein part consists of a Cas9 nuclease (nCas9) and a reverse transcriptase domain, and its pegRNA consists of the guide RNA sequence and additional sequences, including the primer binding site, reverse transcription (RT) template, and the edit sequence (**A**,**B**). After the nick is created, the RT is initiated by the reverse transcriptase to introduce the edit sequence into the 3′ flap end at the target site (**C**), followed by additional steps to incorporate the edit into the genome (**D**–**F**). * Modified from reference [15,16] and created in BioRender. Xu, J. (2025) https://BioRender.com/wlcvm8j (accessed on 1 December 2025).

One limitation with base editors is that they can only install point mutations, but not other types of edits, for example, insertions or deletions. The PRIME editors are developed to address such needs [14,28]. Like base editors, PRIME editors utilize nuclease-defective Cas9, in most cases Cas9 nickase, to avoid generating DSBs. Unlike base editors, the PRIME editor’s fusion partner is a reverse transcriptase domain, and instead of using the sgRNA, as in Cas9 and Cas9 base editors, PRIME editors use a pegRNA. The pegRNA contains the guide RNA sequence to locate the complex to the target sequence; it also contains a reverse transcription (RT) template to introduce the edit, and a primer binding site (PBS) sequence where the reverse transcription initiates. PRIME editors can achieve versatile editing types, including insertion, deletion, and point mutation, as long as such desirable changes are coded in the RT template. Figure 4 illustrates the PRIME editor working mechanism.

## 3. GETx Strategies

**Figure 5:**
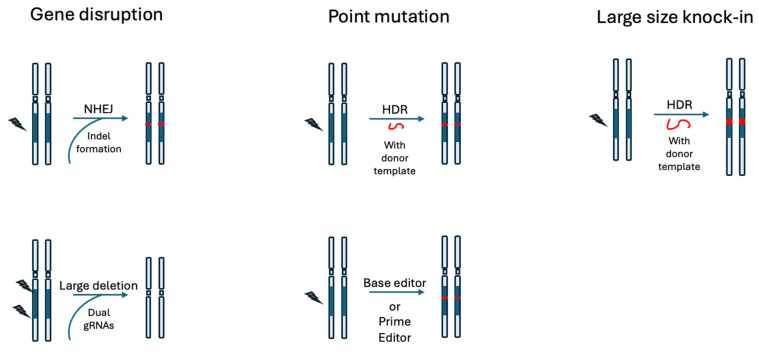
Illustration of therapeutic strategies: gene disruption, point mutation, and large-scale gene knock-in. (**Left**): Gene disruption is often created by an indel mutation (**top**) or large deletion (**bottom**). (**Middle**): Point mutation is often created by HDR-mediated mutation (**top**) or by base editor or prime editor (**bottom**). (**Right**): Large-scale gene knock-in is often created by HDR-mediated knock-in.

The application of gene editing tools to cure or treat human diseases is referred to as GETx in this review. GETx can be broadly classified into three main categories (Figure 5) based on the specific editing type it exploits [29], each tailored to address different genetic etiologies and clinical needs: (i) loss-of-function editing; (ii) point mutation correction; and (iii) large-scale gene knock-in. In the context of LivGETx-CVD, the first two are of more relevance than the third one. This is because, as we shall see later in this review, investigators are mostly targeting genes whose loss-of-function mutation provides cardioprotective effects.

### 3.1. Gene Disruption

This approach inactivates or disrupts the function of a target gene, often to confer disease resistance or eliminate pathogenic gene activity. Clinically, for example, it has been utilized to knock out the C-C motif chemokine receptor 5 (CCR5) gene to provide human immunodeficiency virus (HIV) resistance or to disrupt programmed cell death 1 (PD1) in immune cells to enhance anti-tumor immunity [30,31,32].

Applicable editing strategies to achieve loss-of-function edits include: (i) small insertions or deletions (indels) resulting from NHEJ that lead to frameshift and consequently premature stop codons (this is often achieved by using SpCas9 + sgRNA without any repair templates); (ii) large-scale deletions to remove one or more exons (this can be achieved by using SpCas9 with two or more sgRNAs); (iii) point mutations at the splicing donor or acceptor site to disrupt normal splicing and consequently the reading frame, leading to loss of protein production or function. This can be achieved by using a Cas9 base editor + sgRNA, which we will introduce in a later session.

### 3.2. Point Mutation

Precise point mutation represents a substantial percentage of clinical needs. This strategy is often used to repair pathogenic single-nucleotide variants or small mutations responsible for monogenic disorders. A classic example is the correction of the CFTR-F508del mutation in cystic fibrosis [33,34], restoring normal protein function. Another example is to correct the E6V mutation in the hemoglobin subunit beta (HBB) gene to cure sickle cell disease [35].

Applicable editing strategies to achieve precise point mutation corrections include: (i) HDR, using canonical Cas9 + sgRNA along with double stranded or single stranded DNA donor templates which harbors the correct/function sequence; (ii) base editor + sgRNA to install single-nucleotide substitutions (transition or transversion) for correction; (iii) prime editor + pegRNA to replace the pathogenic sequence in the genome with a repair template coded in the pegRNA.

### 3.3. Large-Scale Gene Knock-In

Knock-in strategies to insert a functional gene, a super exon, or other genetic element at a specific locus in the genome are another GETx strategy. It is often used to provide a “one size fits all” cure to a disease that could result from different types of mutations on the same gene. For example, knocking in a full-length, functional cystic fibrosis transmembrane conductance regulator gene (CFTR) can restore the channel activity and potentially cure the disease, regardless of which particular CFTR mutation a cystic fibrosis (CF) patient carries, which could be one of the several hundred CF-causing mutations [36]. In this way, instead of developing hundreds of mutation-specific GETx drugs, this one large-gene knock-in-based GETx drug would suffice for all.

Applicable editing strategies to achieve precise large-size gene knock-in include: (i) HDR, using canonical Cas9 + sgRNA along with donor templates that harbor the large-size knock-in sequence; (ii) site-specific insertion of large-size gene fragment mediated by transposases, integrases, or recombinases, or other methods, which are well reviewed in these publications [14,37,38].

## 4. Delivery Considerations

How to efficiently and precisely deliver gene editing modalities to the target site in vivo has remained the paramount challenge in GETx. In this session, we provide an overview of current delivery options. Table 1 summarizes the primary features of these systems.

### 4.1. Adeno-Associated Virus

Adeno-associated viruses (AAVs) are non-enveloped viruses with a single-stranded DNA genome, flanked by inverted terminal repeats (ITRs) [44]. The genome contains three main functional genes: Cap, which encodes the capsid proteins responsible for protecting the viral DNA and enabling cell attachment; AAP, which produces an assembly-activating protein supporting capsid assembly; and Rep, which is necessary for viral replication and packaging [45,46].

Since the first AAV-based gene therapy drug, Luxturna, gained approval from the FDA in 2017 [47], AAV has become the most widely used vector for delivering GETx components. This popularity is largely due to its good safety profile, high transduction efficiency to diverse tissues, and low DNA integration into the host genome, typically persisting as an episome [48].

A major disadvantage of AAV is its limited packaging capacity of only 4.7 kb. This challenges the delivery of large gene editors such as SpCas9, base editors, and prime editors that are unable to fit inside a single AAV vector. There are several methods to address the size limitation. The intein-split dual-AAV system is a prominent example, where large gene editing nucleases are split into two halves, fused to split inteins, and delivered in separate AAV vectors [49]. After entry into the same cell, protein splicing by inteins allows reconstitution of the full-length gene editor. This technique has demonstrated robust editing efficiencies across various organs such as the liver, retina, brain, heart, and skeletal muscle [50,51]. In addition, advances in the discovery of smaller Cas orthologs now allow efficient single-AAV delivery. Cas9 variants such as SaCas9 [52], Nme2Cas9 [53], CjCas9 [54], and SauriCas9 [55] are further expanding the toolbox.

Another clinically relevant consideration is patients’ pre-existing immunity to natural AAV serotypes, especially at high doses [56]. To overcome these immune barriers, strategies have been developed, including engineering capsids with reduced immunogenicity, employing immune evasion strategies, and using plasmapheresis to lower neutralizing antibody levels [57,58,59,60].

### 4.2. Lentivirus

As single-stranded RNA viruses, lentiviruses (LVs) carry the enzymes reverse transcriptase, integrase, and protease, which facilitate the reverse transcription and integration of their RNA cargo into the host genome. Due to the large packaging capacity (up to 10 kb), long-term expression, and low immunogenicity, LVs are widely used for ex vivo gene delivery, most notably in hematopoietic stem cells (HSCs) and T cells.

To date, four lentiviral gene therapies have received FDA approval: Zynteglo (for severe β-thalassemia) [61], Skysona (for cerebral adrenoleukodystrophy) [62], Lenmeldy (for early metachromatic leukodystrophy) [63] and Lyfgenia (for sickle cell disease) [64]. They all utilized ex vivo modification of patient-derived HSCs followed by reinfusion. Additionally, several FDA-approved CAR-T therapies, including Tisagenlecleucel [65] for leukemia, and Axicabtagene ciloleucel [66], Brexucabtagene autoleucel [67], and Lisocabtagene maraleucel [68] for lymphomas, use lentiviral vectors for gene delivery to T cells.

The ability of lentiviruses to integrate their genetic payload into the host genome is highly advantageous for ex vivo applications because it ensures stable and long-term gene expression. However, this same characteristic poses safety concerns for in vivo use, as integration may result in insertional mutagenesis or off-target effects. To address these risks, integrase-deficient lentiviral vectors (IDLVs) have been engineered so that the delivered genetic material persists episomally rather than integrating into the host DNA [69]. While IDLVs have significantly lower integration rates, they are not entirely free from residual integration events and can still drive prolonged transgene expression [70], which limits the use of LVs for in vivo gene editing applications.

### 4.3. Adenovirus

Adenoviruses (Adv) are non-enveloped viruses with a double-stranded DNA genome. They have large cargo capacity (8–9 kb), high transduction efficiency, transient gene expression, and low integration into the host genome. Owing to these advantages, Adv vectors have been employed for in vivo gene delivery since the early 1990s, with initial studies targeting alpha-1 antitrypsin deficiency [71] and cystic fibrosis [72].

However, the clinical development of Adv gene therapies has been significantly hampered by safety concerns. Notably, a 1993 clinical trial for ornithine transcarbamylase (OTC) deficiency resulted in fatal multisystem organ failure in one participant, and other subjects experienced serious complications, such as acute elevations in hepatic transaminase, hypophosphatemia, and thrombocytopenia [73]. To address these risks, researchers are investigating several strategies. For example, administration of anti-inflammatory agents such as dexamethasone can ease inflammatory responses and increase tolerance to the vector [74]. Chemical modification of the viral capsid, for instance, by attaching synthetic polymers [75], can help shield the virus from immune detection. Furthermore, genetic alterations of the capsid structure [76] can reduce immune recognition and enable more durable expression of the therapeutic gene.

### 4.4. Lipid Nanoparticles

Lipid nanoparticles (LNPs) have emerged as a widely used nonviral vector for gene editing agents. LNPs are fully synthetic and generally constructed from four components: (i) ionizable cationic lipids are critical both for encapsulating negatively charged cargos through electrostatic interactions and facilitating endosomal escape; (ii) helper phospholipids are mostly found in the outer layer and enhance structural stability, nanoparticle integrity and membrane interactions; (iii) cholesterol fills gaps between phospholipids, boosting nanoparticle stability, integrity, and membrane fusion during cell entry and intracellular trafficking; and (iv) PEG-lipids are coated on nanoparticle surface, reducing aggregation, improving particle stability and half-life and lowering immunogenicity [77].

LNPs can encapsulate and deliver DNA, RNA, and protein cargos, with RNA delivery being the most established. LNPs deliver their payloads by entering cells via endocytosis, after which cargo release into the cytosol relies primarily on endosomal escape [78].

LNPs provide several advantages over viral vectors for GETx delivery, which include the transient expression duration and the flexibility in cargo size. Further, most LNP components are biodegradable and well-tolerated in vivo. However, a notable challenge remains: the efficiency of endosomal escape remains low, as cargos trapped in endosomes are often destined for lysosomal degradation [78]. To overcome this, ongoing research explores optimized particle compositions, surface modifications by adding cell-penetrating peptides [79] and external triggers (like light or ultrasound) to trigger cargo release at the desired time [80].

Clinically, LNPs have enabled major advances, including the FDA-approved siRNA therapy for hereditary transthyretin (ATTR) amyloidosis [81], and the mRNA COVID-19 vaccines from Pfizer-BioNTech and Moderna [82,83]. Ongoing trials using LNPs for gene editing therapies are summarized in Table 2.

### 4.5. Virus-Like Particles

Engineered virus-like particles (eVLPs) are constructed from retroviral capsid proteins, but they do not contain the viral genome, which makes them non-replicative and incapable of integrating into the host DNA, two desirable features in GETx. Typically, the viral capsid structural framework is provided by proteins such as Gag. To hijack the system to deliver GETx components, Gag can be fused with the desired cargo (e.g., Cas9 base editor) via a linker that is cleavable by protease, ensuring cargo release once the particle matures. The tissue specificity of eVLPs is determined by envelope glycoproteins like VSV-G for broad tropism or FuG-B2 for neuron-specific targeting [84]. Therefore, eVLPs utilize the high efficiency and precise tissue targeting advantages of viral vectors but avoid the risk of insertional mutagenesis and persistent transgene expression that can occur with conventional viral vectors [80].

The therapeutic promise of this technology has been demonstrated in animal studies [85,86]. Despite these significant advantages, the clinical translation of eVLPs still faces barriers, primarily due to the fact that large-scale manufacturing and batch-to-batch particle stability can be difficult. Plus, their viral protein origin raises some concerns about immunogenicity. To address these issues, researchers are engineering the capsid protein [87], optimizing purification processes [88] and using human-derived gag-like structural protein, such as PEG10, to decrease immune responses [89].

## 5. Genes of Interest in LivGETx-CVD

**Figure 6:**
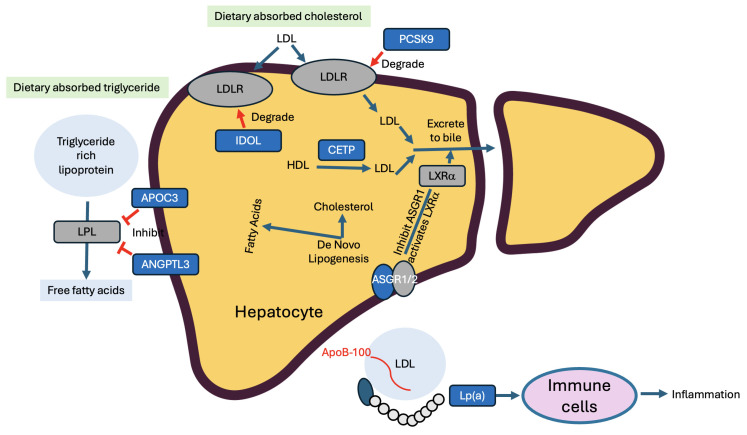
Illustration of key targets and their mechanistic pathways in LivGETx-CVD. One focal point is LDLR, which plays a central role in cholesterol metabolism. PCSK9 and IDOL contribute to the degradation of LDLR. Therefore, targeting PCSK9 or IDOL is expected to reduce the degradation of LDLR and consequently reduce LDL-C levels. CETP and ASGR1 are also involved in LDL-C metabolism: CETP moves cholesteryl esters from HDL to LDL in exchange for triglycerides, thereby regulating cholesterol levels, and ASGR1 regulates hepatic cholesterol excretion, and inhibition of ASGR1 is shown to promote cholesterol excretion. Therefore, targeting CETP or ASGR1 is expected to increase HDL-C and reduce LDL-C levels. Another focal point is LPL, which breaks down TG to free fatty acids. APOC3 and ANGPTL3 both inhibit LPL’s activity. Therefore, targeting APOC3 or ANGPTL3 is expected to enhance LPL activity and reduce TG levels. Lp(a) plays a role that is not directly involved in cholesterol or TG metabolism. Rather, its existence in the LDL particle is associated with enhanced inflammatory effects. Targeting LPA is expected to reduce inflammation, which is another risk factor for atherosclerosis and CVD.

In this section, we focus specifically on liver genes regulating lipid metabolism, with the aim of modulating these targets to promote healthier lipid profiles, thereby decreasing atherosclerosis risk and ultimately reducing the incidence of cardiovascular disease (Figure 6 and Table 2). We present the rationale why targeting these genes may render benefits to the patients, followed by preclinical and clinical supporting evidence, snapshots of ongoing LivGETx-CVD clinical trials targeting these genes (as of 1 November 2025, summarized in Table 3), as well as risk considerations.

### 5.1. PCSK9

#### 5.1.1. Rationale

Proprotein convertase subtilisin/kexin type 9 (PCSK9) regulates circulating cholesterol levels by modulating the number of LDL receptors (LDLR) on the hepatic cell surface. When intracellular cholesterol decreases, transcriptional activity of sterol regulatory element-binding protein 2 (SREBP2) increases, which upregulates both PCSK9 and LDLR expression. PCSK9 is secreted and subsequently binds to LDLR, leading to lysosomal degradation of the receptor. This reduces hepatic LDLR availability and consequently decreases hepatic clearance of LDL from the bloodstream [90,91]. Based on this knowledge, it is hypothesized that PCSK9 loss of function will lead to reduced cholesterol levels (see below).

*PCSK9 loss of function → retention of LDLR → reduced cholesterol in the blood→ less CVD risks*

Human population genetics studies have shown that individuals carrying natural loss-of-function alleles in *PCSK9* have significantly lower LDL cholesterol levels throughout life and a reduced risk of coronary heart disease and major cardiovascular events compared to non-carriers [92]. Large cohort studies and meta-analyses demonstrate that these variants are associated with up to a 50% reduction in coronary heart disease risk, providing strong genetic evidence that lifelong inhibition of PCSK9 lowers cardiovascular risk in humans [93].

#### 5.1.2. Preclinical Supporting Evidence

In 2014, Ding et al. employed an all-in-one Adv vector to deliver Cas9 and sgRNA targeting *PCSK9* exon 1 in mice, achieving over 50% mutagenesis and a 40% reduction in LDLC levels after four days [94]. Application to humanized mouse livers yielded a 52% reduction in human PCSK9 [95]. In 2017, Chadwick et al. utilized a base editor (BE3) delivered by an Adv vector, which reduced PCSK9 expression by more than 50% and decreased cholesterol levels by approximately 30% in mice [96]. Later, AAV delivered smaller Cas9 variants, saCas9, enabling up to 95% PCSK9 knockdown and 40% reduction in total cholesterol in mice [97]. David Liu’s group further overcame AAV capacity constraints by delivering intein split base editors on dual AAVs, achieving comparable editing as all-in-one systems [50]. As prolonged Cas9 expression can cause off-target effects, a self-cleaving AAV-CRISPR-Cas9 system was developed to minimize these risks without sacrificing gene editing efficiency [98]. VLPs have also been used to deliver base editors to target *PCSK9*, achieving a 78% knockdown of PCSK9 and 63% editing efficiency in the mouse liver [85].

In 2024, it is reported that a single injection of LNP-formulated mRNAs encoding epigenetic editors resulted in up to 75% inhibition of circulating PCSK9 for almost one year in mice [99]. Notably, gene silencing and epigenetic repressive effects at the *PCSK9* locus persisted even after liver regeneration, supporting the heritability of this epigenetic modification [99]. Another epigenetic editor designed to methylate the human *PCSK9* locus and delivered via LNP achieved near-complete and durable PCSK9 silencing in humanized transgenic mice for at least one year. Remarkably, reversibility was demonstrated using an activator to demethylate previously silenced PCSK9, underscoring the potential of this approach for both durable and reversible gene silencing [100].

Translation into large animal models includes AAV–meganuclease-mediated *PCSK9* targeting in rhesus macaques, a non-human primate (NHP) model species, which produced an 84% reduction in PCSK9 and a 60% decrease in LDL, though off-target and immune responses were observed [101]. In a subsequent study, LNP-delivered ABE mRNA resulted in near-complete PCSK9 knockdown and a 60% reduction in LDL in another NHP species, Macaca fascicularis, with minimal off-target effects [102]. Another study demonstrated that the same strategy achieved stable reductions in PCSK9 (32%) and LDL (14%) in macaques [103]. In yet another NHP study, a single intravenous dose of AAV injection is well tolerated and lowers PCSK9 by 83% and LDL-C by 69% for up to 476 days, with no germline tissue distribution [104].

In 2025, a split prime editor architecture (split-PE-367 with Rma intein) was developed. When packaged in AAV9, it achieved 17.5% precise editing at the *PCSK9* locus in mice [105]. This work established new split sites to enable effective and robust prime-editing strategies in LivGETx-CVD.

#### 5.1.3. Clinical Supporting Evidence (Other than LivGETx-CVD)

Clinical trials have shown that PCSK9 inhibitors reliably lower plasma LDL levels. Currently, there are two FDA-approved PCSK9 monoclonal antibodies: alirocumab [106] and evolocumab [107]; there is also one approved siRNA: inclisiran [108]. Other candidates progressing through clinical trials include monoclonal antibodies (ongericimab [109], tafolecimab [110], ebronucimab [111], and recaticimab [112]), anti-sense oligonucleotide (ASO) (AZD8233 [113]), siRNA (RBD7022 [114]), adnectin (Lerodalcibep [115]), oral peptides (MK-0616 [116] and NNC0385-0434 [117]), and small molecule (AZD0780 [118]).

#### 5.1.4. Ongoing LivGETx-CVD Clinical Trials Targeting *PCSK9*

***VERVE-101*** is developed by Verve Therapeutics, designed to treat cardiovascular disease by targeting the *PCSK9* gene in the liver. The therapy uses an adenine base editor (ABE), which makes a precise A-to-G base change at the *PCSK9*, resulting in a loss-of-function mutation that shuts down PCSK9 protein production. VERVE-101 is delivered via LNPs specifically targeted to hepatocytes. The trial is currently in Phase 1b (Heart-1), but enrollment was paused due to safety concerns, including transient liver enzyme elevations and thrombocytopenia in some participants, which were attributed to the LNP delivery system rather than the gene editor itself. Early results show dose-dependent reductions in PCSK9 protein (up to 84%) and LDL cholesterol (up to 55%) that have been durable for at least 180 days, with mean LDL-C reductions of 42–57% in higher-dose cohorts. The trial remains paused while Verve investigates the safety profile and advances a successor therapy, VERVE-102 (see below), with an improved LNP formulation [119].

***VERVE-102*** is developed by Verve Therapeutics and is currently in Phase 1b clinical trials (the Heart-2 study) for patients with heterozygous familial hypercholesterolemia (HeFH) and/or premature coronary artery disease. Like its predecessor VERVE-101, VERVE-102 uses ABE to make an A-to-G transition without double-stranded breaks to inactivate the *PCSK9* gene in hepatocytes. VERVE-102 is delivered as an mRNA and guide RNA packaged in LNPs for liver targeting. As of April 2025, VERVE-102 showed promising interim results: a mean 53% reduction in LDL cholesterol across dose cohorts, with the highest cohort achieving up to 69% LDL-C reduction and up to 84% reduction in PCSK9 levels; the safety profile has been favorable, with no treatment-related serious adverse events. Triglyceride effects are not prominently reported, as focus remains on LDL-C and PCSK9; the trial is ongoing, with plans to launch Phase 2 after regulatory clearance, supported by involvement from Eli Lilly as a collaborator.

***ART002*** is developed by AccurEdit Therapeutics for the treatment of heterozygous familial hypercholesterolemia (HeFH). The therapy uses a canonical Cas9 mRNA and a single-guide RNA, delivered to the liver via LNPs. ART002 creates a loss-of-function mutation in the *PCSK9* gene by silencing its expression, resulting in sustained reductions in LDL cholesterol levels. The therapy is administered as a single intravenous dose, and clinical trial data from a multicenter, open-label, single-arm investigator-initiated trial in China (now completed) demonstrated that a single dose led to pharmacological saturation, significant (>50%) reductions in LDL-C, and favorable safety with no serious adverse events reported [120].

***YOLT-101*** is developed by YolTech Therapeutics, currently in a Phase 1 investigator-initiated clinical trial in China, and cleared for IND-enabling trials in both China and the US for the treatment of heterozygous familial hypercholesterolemia (HeFH). The therapy uses YolTech’s proprietary adenine base editor (hpABE5), composed of a Cas9 nickase (nCas9) fused to a deaminase derived from Hafnia paralvei, enabling precise A-to-G base conversion within the target gene *PCSK9*. YOLT-101 creates a loss-of-function mutation in the *PCSK9* gene, providing long-lasting reductions in LDL cholesterol. Delivery is achieved via LNPs administered by intravenous infusion targeting hepatocytes in the liver. Interim results from six subjects in the highest dose group (0.6 mg/kg) revealed a mean 50% LDL cholesterol reduction and more than 70% decrease in PCSK9 protein at 4 months post-dose, with no serious adverse events and only mild, transient infusion-related or liver enzyme elevations reported; effects on triglycerides have not been prominently reported.

#### 5.1.5. Risk Considerations

One potential risk of gene editing *PCSK9* to prevent cardiovascular disease is that complete loss of PCSK9, although unlikely, could result in very low LDL cholesterol levels, which may impair the body’s ability to maintain adequate cholesterol supply for hormone synthesis and cell membrane function. Lifelong loss of PCSK9 function is generally well tolerated, but extremely low LDL levels may increase the risk of developing diabetes in some individuals due to altered lipid signaling affecting glucose metabolism [121]. PCSK9 also has roles in immunity and modulating viral infection risk, so its absence could potentially weaken innate immune responses or increase susceptibility to certain pathogens [122]. Since PCSK9 influences lipid homeostasis in the brain, chronic deficiency might have subtle effects on neurocognitive function, though evidence in humans is still limited [123].

### 5.2. ANGPTL3

#### 5.2.1. Rationale

Angiopoietin-like protein 3 (ANGPTL3) is a critical regulator of plasma triglyceride and cholesterol metabolism, primarily through inhibition of lipoprotein lipase (LPL) and endothelial lipase (EL) activity [124]. Consequently, loss of ANGPTL3 function is hypothesized—and has been proved in both human genetic studies and animal models—to reduce circulating triglyceride and LDL-C levels (see below).

*ANGPTL3 loss-of-function → less inhibition of LPL → increased digestion of triglycerides → reduced triglyceride levels in the blood → less CVD risk*

Individuals with heterozygous loss-of-function mutations in *ANGPTL3* exhibit reduced triglycerides, HDL-C, LDL-C, and a lower risk of cardiovascular disease [125]. Moreover, individuals in the lowest tertile of circulating ANGPTL3 protein concentrations have significantly reduced risks of myocardial infarction, confirming that reduced ANGPTL3 activity confers natural protection against atherosclerotic cardiovascular disease [126].

#### 5.2.2. Preclinical Supporting Evidence

Gene editing has demonstrated strong potential for ANGPTL3 inhibition. In a study by Chadwick et al., an adenoviral base editor (BE3) targeting *ANGPTL3* in C57BL/6J mice led to reductions in plasma ANGPTL3 (49%), triglycerides (31%), and total cholesterol (19%), with even higher efficacy in LDLR knockout mice [127]. ANGPTL3-targeted therapies predominantly lower triglycerides, whereas PCSK9 inhibition is more effective for LDL lowering; combined targeting provided no additive benefit [127].

CRISPR/Cas9 delivery via LNP also enabled efficient, liver-specific silencing of ANGPTL3 in mice, significantly reducing LDL-C and triglyceride levels without off-target effects or liver toxicity [128]. Similarly, dual AAV delivered intein split CBE in mice nearly eliminated all circulating ANGPTL3 and lowered triglycerides and total cholesterol by 58% and 61%, respectively [129]. Single-AAV delivery of a mini ABE achieved robust dual knockdown of PCSK9 and ANGPTL3 (>90%) and markedly reduced cholesterol [130]. LNP-delivered ABE mRNA targeting an *ANGPTL3* splice site in mice achieved over 60% liver editing and sustained reductions in ANGPTL3, LDL-C, and triglycerides for at least 100 days. Lipid levels remained low after 191 days when challenged with a high cholesterol diet [131].

#### 5.2.3. Clinical Supporting Evidence (Other than LivGETx-CVD)

A variety of strategies are being explored to target ANGPTL3. Evinacumab, a monoclonal antibody, was already approved in 2021 for managing homozygous familial hypercholesterolemia. It has shown impressive reductions in both LDL cholesterol by 50% and triglycerides by 55% in clinical trials [132,133,134]. In addition, Arrowhead’s siRNA drug ARO-ANG3 [135] and Lilly’s siRNA agent, solbinsiran [136] have both reached Phase 2 studies with significantly decreased triglycerides and LDL-C. However, the antisense oligonucleotide, vupanorsen, was discontinued for safety concerns [137].

#### 5.2.4. Ongoing LivGETx-CVD Clinical Trials Targeting *ANGPTL3*

***VERVE-201*** is developed by Verve Therapeutics, utilizing an adenine base editor (ABE) system to precisely install a loss-of-function mutation in the *ANGPTL3* gene. The therapy is delivered by Verve’s proprietary ligand-targeted GalNAc-lipid nanoparticle (GalNAc-LNP) system, enabling efficient and LDLR-independent liver targeting via intravenous infusion. VERVE-201 is being tested in the ongoing open-label Phase 1b Pulse-1 clinical trial for adults with refractory hypercholesterolemia (RH) and homozygous familial hypercholesterolemia (HoFH), aiming to achieve long-term reduction in LDL cholesterol, triglyceride-rich lipoproteins, and blood ANGPTL3 protein. Detailed quantitative clinical trial results are anticipated in late 2025; safety signals remain favorable, and the trial has not been terminated.

***CTX310*** is developed by CRISPR Therapeutics for the treatment of dyslipidemias such as familial hypercholesterolemia and severe hypertriglyceridemia. The technology utilizes a canonical CRISPR-Cas9 nuclease, delivered as mRNA with a guide RNA, targeting the *ANGPTL3* gene for direct gene disruption. By creating double-strand breaks, CTX310 induces a loss-of-function mutation in *ANGPTL3* to reduce circulating triglycerides and LDL cholesterol. The therapy is administered via a single intravenous infusion, using LNPs to direct liver-specific editing. The recently published (November 2025) Phase 1 trial results demonstrated robust, dose-dependent lipid-lowering effects in adults with severe or refractory dyslipidemia [138]. At the highest dose tested (0.8 mg/kg), the therapy produced mean reductions from baseline of 73% in circulating ANGPTL3 protein (maximum 89%), 55% in triglycerides (maximum 84%), and 49% in LDL cholesterol (maximum 87%). Safety was favorable, with no treatment-related serious adverse events and no greater than grade 3 liver enzyme elevations.

#### 5.2.5. Risk Considerations

Gene editing *ANGPTL3* to prevent cardiovascular disease may cause excessive lowering of triglycerides and cholesterol. There has been evidence showing that a deficiency in ANGPTL3 is associated with familial combined hypolipidemia [139]. In addition, ANGPTL3 is important for lipid partitioning and regulation of lipase activity in multiple tissues, so its absence could disrupt normal fat metabolism and lead to hepatic steatosis or abnormal fat accumulation in the liver [140].

### 5.3. CETP

#### 5.3.1. Rationale

CETP plays a crucial role in lipoprotein metabolism by mediating the transfer of cholesteryl esters, triglycerides, and phospholipids between HDL, LDL, and VLDL, thereby contributing to reverse cholesterol transport from peripheral tissues to the liver [141]. Since high levels of HDL-C are inversely associated with cardiovascular disease risk, raising HDL-C through CETP inhibition has been explored as a therapeutic strategy [142]. It is therefore hypothesized that CETP loss-of-function will lead to increased HDL-C levels and decreased LDL-C levels (see below).

*CETP loss-of-function → less conversion of HDL to LDL → increased HDL-C and decreased LDL-C levels in the blood → less CVD risk*

Recent large cohort studies indicate that people with genetic *CETP* deficiency have lower non-HDL cholesterol, higher HDL cholesterol, and an overall antiatherogenic lipid profile. In particular, a Danish population-based study found that genetic *CETP* deficiency was associated with reduced risks for cardiovascular death, ischemic heart disease, myocardial infarction, and peripheral artery disease [143]. However, some studies in other populations (such as Chinese adults) found increased HDL but little or no reduction in CVD risk unless non-HDL cholesterol was also lowered [144]. Overall, population genetics provides evidence that CETP loss-of-function confers reduced CVD risk when it leads to lower non-HDL cholesterol as well as higher HDL cholesterol.

#### 5.3.2. Preclinical Supporting Evidence

Our group generated CETP knockout (KO) rabbits using zinc-finger nuclease, which exhibit increased HDL-C, decreased total cholesterol, and significantly reduced development of diet-induced atherosclerosis compared to wild-type controls [145]. This study demonstrates that in vivo *CETP* gene editing offers a promising approach to cardiovascular disease prevention.

#### 5.3.3. Clinical Supporting Evidence

Current treatments, such as antisense oligonucleotides [146] and small-molecule inhibitors, have shown the ability to raise plasma HDL and reduce atherosclerosis in preclinical models. However, translation of CETP inhibition to humans has been challenging. Early CETP inhibitors, including torcetrapib [147], dalcetrapib [148], Anacetrapib [149], and evacetrapib [150], were either discontinued in clinical trials for failure to demonstrate reduced CVD risk or severe adverse effects, or lacked commercial potential. Obicetrapib is the only candidate in the ongoing Phase III trial and exhibited significant elevation of HDL, reduction in LDL, and Lp(a) with a tolerated safety profile [151].

#### 5.3.4. Risk Considerations

Gene editing *CETP* to prevent cardiovascular disease may lead to marked increases in HDL cholesterol, but could also result in impaired reverse cholesterol transport. This disruption might paradoxically affect the ability to clear cholesterol from arterial walls, which could limit or even negate the expected protection against atherosclerosis in some individuals. Also, when CETP is inhibited, the resulting HDL particles tend to be more enriched in cholesteryl esters and may lose their protective effects against atherosclerosis [152]. Additionally, CETP deficiency has been associated with an increased risk of age-related macular degeneration and retinal abnormalities, raising concerns about eye health with long-term CETP inhibition [143]. Lastly, the failures of CETP small-molecule inhibitor drugs ring a loud alarm to any new drug development targeting CETP.

### 5.4. ApoC3

#### 5.4.1. Rationale

Apolipoprotein C3 (ApoC3) is a small apolipoprotein primarily produced in the liver and, to a lesser extent, in the small intestine. It plays a pivotal role in triglyceride-rich lipoprotein (TRL) metabolism. Its actions are mediated through three main mechanisms: (1) inhibition of LPL-mediated lipolysis of TRLs [153,154], (2) suppression of TRL clearance by interfering with the binding of ApoB and ApoE to hepatic lipoprotein receptors [155], and (3) promotion of VLDL assembly and secretion under lipid-rich conditions [156]. It is therefore hypothesized that ApoC3 loss of function will result in substantially reduced triglyceride levels (see below).

*ApoC3 loss-of-function → less inhibition of LPL → increased digestion of triglycerides → reduced triglyceride levels in the blood → less CVD risk*

Studies in human populations have demonstrated an association between ApoC3 and cardiovascular diseases. Individuals carrying loss-of-function mutations in *ApoC3* exhibit lower plasma triglyceride levels and a substantially reduced risk of coronary heart disease [157], and even heterozygous carriers tend to have reduced triglyceride compared to non-carriers [158].

#### 5.4.2. Preclinical Supporting Evidence

CRISPR/Cas9 technology has been used to generate ApoC3 knockout animal models to advance dyslipidemia research. Although overexpression of human ApoC3 greatly accelerates atherosclerosis in mice, interestingly, ApoC3 deficiency does not confer atherosclerosis protection in this species. To address this, researchers created an ApoC3 knockout hamster. Compared to mice, hamsters exhibit lipid metabolism and lipoprotein profiles more similar to humans. This model revealed a direct correlation between ApoC3 and atherogenesis [159]. Likewise, ApoC3 knockout rabbits generated via ZFN with an adenine insertion exhibited lower triglyceride and reduced atherosclerosis on both chow and Western diets, as well as accelerated clearance of β-VLDL, a major atherogenic lipoprotein [160]. These findings suggest that inhibition of ApoC3 not only holds promise for the treatment of hypertriglyceridemia but may also be a therapeutic strategy for atherosclerosis. Expanding on these findings, Scribe Therapeutics reported that its LNP-delivered, CRISPR/CasX-based lead molecule STX1400 achieved more than 90% ApoC3 knockdown and produced marked reductions in triglyceride level in a humanized mouse model [161].

While not a primary focus of this review, it is worth pointing out that mouse models do not always model well lipid metabolism, atherosclerosis, and cardiovascular diseases. Non-murine model species, for example, hamster, rabbit, pig, and NHPs, may serve as alternative models that better recapitulate human lipid and CVD pathophysiology.

#### 5.4.3. Clinical Supporting Evidence (Other than LivGETx-CVD)

ApoC3 has gained attention as a promising target for reducing triglyceride levels and cardiovascular disease risk. Volanesorsen is an early-generation ApoC3-inhibiting ASO drug. It exhibited triglycerides reduction of up to 77% in phase 3 trials [162] and has been approved in Europe. However, Volanesorsen faced regulatory hurdles in the US due to the risk of thrombocytopenia [163]. Olezarsen, the GalNAc-conjugated ASO, has shown significant efficacy in lowering triglycerides by 44% [164]. It was approved by the FDA in 2025 for patients with familial chylomicronemia syndrome (FCS). In addition, GalNAc-conjugated siRNA plozasiran has demonstrated sustained reductions in triglycerides by 62% and is currently under Phase III trials [165]. Other clinical testing of siRNA drugs includes RBD-5044 [166] and RN0361 [162], both in Phase II. There are no monoclonal antibody drugs targeting ApoC3 at present, but several candidates are in the preclinical stage [167,168].

#### 5.4.4. LivGETx-CVD Clinical Trials Targeting *ApoC3*

***CS121*** is developed by CorrectSequence Therapeutics for treating familial chylomicronemia syndrome (FCS) and severe hypertriglyceridemia. The therapy uses Correctseq’s proprietary next-generation transformer base editor (tBE), which enables precise single-base A-to-G transitions at the *APOC3* gene without creating DNA double-strand breaks. CS121’s mechanism mimics natural loss-of-function variants of APOC3, delivering a sustained reduction in plasma triglyceride levels by permanently downregulating APOC3 expression. The therapeutic components are delivered intravenously using LNPs for liver-targeted editing. As of November 2025, CS121 is in a Phase 1, open-label, single-arm, adaptive dose-escalation clinical trial; the first treated patient saw fasting triglycerides drop significantly within three days post-treatment, was discharged without any adverse events, and longer-term follow-up for safety, PK/PD, and pancreatitis outcomes is ongoing.

#### 5.4.5. Risk Considerations

Since ApoC3 inhibits lipoprotein lipase, its loss might lead to excessively rapid clearance of triglycerides, potentially disrupt energy reserve in the body, and cause metabolic disorders. Loss of triglycerides could also alter the function of remnant particles involved in fat-soluble vitamin transport, with possible impacts on vitamin absorption [169]. ApoC3 also inhibits hepatic uptake of TG-rich lipoprotein (TRL) remnants, so its deficiency may lead to hepatic steatosis or abnormal fat deposition [170].

### 5.5. ASGR1

#### 5.5.1. Rationale

*ASGR1* encodes a subunit of the asialoglycoprotein receptor, which is responsible for mediating the endocytosis and degradation of desialylated proteins in the bloodstream [171,172]. Blocking or mutating ASGR1 disrupts the internalization and lysosomal breakdown of asialoglycoproteins, which in turn suppresses mTORC1 signaling and activates AMPK. Activated AMPK destabilizes the BRCA1/BARD1 complex and increases LXRα, leading to the upregulation of cholesterol transporters such as ABCA1 and ABCG5/G8. With more ABCG5/G8, cholesterol is more efficiently pumped into bile and eliminated from the body through feces [173]. This mechanism suggests that inhibiting ASGR1 could promote cholesterol clearance and be a promising strategy for treating hyperlipidemia. It is therefore hypothesized that ASGR1 loss-of-function will lead to reduced cholesterol levels (see below).

*ASGR1 loss-of-function → upregulation of reverse cholesterol transport → increased cholesterol excretion through feces → reduced cholesterol levels → less CVD risk*

In 2016, a study published in *The New England Journal of Medicine* reported that a loss-of-function variant in ASGR1 was associated with lower non-HDL cholesterol levels and reduced risks of atherosclerosis in humans [174]. Studies of large Icelandic cohorts identified individuals carrying the del12 deletion variants in ASGR1, who have a 34% lower risk of coronary artery disease compared to non-carriers [174].

#### 5.5.2. Preclinical Supporting Evidence

Similar findings were observed in ASGR1 knockout pigs, which serve as a useful model due to their cardiovascular system similarities to humans. These pigs had lower non-HDL cholesterol and developed fewer atherosclerotic lesions after six months on a high-cholesterol diet [175]. Targeting *ASGR1* was further validated in mouse models, where ASGR1 knockout led to reduced blood cholesterol and increased cholesterol excretion into bile [173].

#### 5.5.3. Risk Considerations

Gene editing *ASGR1* to prevent cardiovascular disease may interfere with the liver’s ability to clear asialoglycoproteins, which could lead to impaired liver detoxification and accumulation of glycoproteins. ASGR1 deficiency has been shown to increase susceptibility to liver injury in chronic or acute liver injury mouse models. And ASGR1 deficiency-mediated liver injury was identified to be associated with increasing GP73-mediated hepatic endoplasmic reticulum stress [176]. However, ASGR1 KO mice showed no liver toxicity under dietary challenge in Wang et al.’s *Nature* paper [173].

### 5.6. LPA

#### 5.6.1. Rationale

Lipoprotein(a) is a complex molecule consisting of apolipoprotein(a), encoded by the *LPA* gene, covalently attached to apolipoprotein B100 (ApoB100). Elevated Lp(a) levels are recognized as an independent, causal, and largely genetic risk factor for atherosclerosis, primarily due to mechanisms involving enhanced atherogenesis, inflammation, and thrombosis [177].

It is therefore hypothesized that Lp(a) loss-of-function will lead to reduced inflammation levels and provide benefits to CVD (see below).

*Lp(a) loss-of-function → less inflammation → less CVD risk*

Numerous large-scale epidemiological and Mendelian randomization studies have found that individuals with genetic variants resulting in lower *LPA* gene expression or reduced Lp(a) levels have substantially lower risk of coronary heart disease, aortic valve stenosis, stroke, and other cardiovascular outcomes [178,179]. These clinical studies provided strong genetic evidence that *LPA* inhibition is beneficial for cardiovascular disease prevention.

#### 5.6.2. Preclinical Supporting Evidence

Gene editing strategies targeting *LPA* are under active investigation. Doerfler et al. developed *LPA* transgenic mice (as rodents do not have the *LPA* gene) and disrupted the gene using AAV-delivered saCas9. This resulted in nearly complete knockdown of apo(a) protein as measured by ELISA, although total cholesterol levels remained unchanged in both genders [180]. In 2025, another approach utilized TALEN mRNAs encapsulated in LUNAR^®^ LNPs, administered to mice carrying a human *LPA* transgene. A single treatment reduced plasma Lp(a) levels by more than 80%, persisting for at least five weeks [181]. Although hamsters, rabbits, and pigs exhibit lipid metabolism and lipoprotein profiles more similar to humans compared to mice, none of them naturally express the *LPA* gene and produce Lp(a). Therefore, studies involving *LPA* will need to use transgenic approaches to introduce the human *LPA* gene when modeling Lp(a) biology, as with mice. Because non-human primates (NHP) share closer genetic and physiological similarities to humans, including natural expression of *LPA*, they serve as valuable models for studying Lp(a) biology and therapies targeting the *LPA* gene.

The gene editing therapy CTX320™ involves encapsulating Cas9 mRNA and its guide RNA in LNPs for targeted delivery. In cynomolgus monkeys, a single CTX320™ infusion produced 94% reduction in plasma Lp(a) sustained through Day 224. CTX320™ has now advanced into clinical trials [182].

#### 5.6.3. Clinical Supporting Evidence (Other than LivGETx-CVD)

Several therapeutic interventions are being explored to lower Lp(a). Pelacarsen is an antisense oligonucleotide (ASO) therapy currently in Phase III trials with Lp(a) reduction by 92% [183]; it works by reducing hepatic synthesis of apo(a) in patients with high Lp(a) level [184]. Similarly, siRNA-based drugs have shown remarkable efficacy. Olpasiran, Lepodisiran, and Zerlasiran achieved reductions in Lp(a) of almost 100% and have all entered Phase III [184,185,186,187]. The Lp(a) oral inhibitor LY3473329 has completed Phase II [188,189].

#### 5.6.4. Ongoing LivGETx-CVD Clinical Trials Targeting *LPA*

***CTX320*** is developed by CRISPR Therapeutics, currently in a Phase 1 clinical trial targeting the *LPA* gene, which encodes apolipoprotein(a), to treat patients with elevated Lp(a) and atherosclerotic cardiovascular disease or aortic valve stenosis. The therapy uses canonical Cas9 mRNA with a guide RNA, delivered to the liver via LNPs through a single intravenous infusion. By targeting and permanently disrupting the *LPA* gene, CTX320 introduces a loss-of-function mutation, leading to durable reductions in plasma Lp(a). As of November 2025, safety is reported as favorable, and the trial remains ongoing with no termination announced. Quantitative clinical results on LDL cholesterol and triglycerides are not yet published, as the main reported outcome to date is Lp(a) reduction, with further updates anticipated.

#### 5.6.5. Risk Considerations

Lp(a) has a role in wound healing and tissue repair through its structural similarity to plasminogen, so reducing it to very low levels could theoretically impair fibrinolysis and healing processes [190]. However, no convincing human data support this in practice. Since Lp(a) functions are not fully understood, a complete or lifelong deficiency could have unforeseen effects on vascular biology and systemic health.

### 5.7. IDOL

#### 5.7.1. Rationale

IDOL, also known as MYLIP, is a widely expressed E3 ubiquitin ligase induced transcriptionally by LXR/RXR in response to increased sterol signaling [191,192]. Three IDOL targets, LDLR, VLDLR, and ApoER2, are intimately related to lipid metabolism [193]. IDOL-mediated ubiquitination promotes their lysosomal degradation. Lack or downregulation of IDOL increases LDLR stability, LDL-C uptake, and protects mice against atherosclerosis, independently of SREBP or PCSK9 [194,195]. Based on this knowledge, it is hypothesized that IDOL loss-of-function will lead to reduced CVD risk (see below).

*IDOL loss-of-function → retention of LDLR → reduced cholesterol → less CVD risk*

Findings of human genetic variants in IDOL support the basic research results, with loss-of-function carriers having very low levels of LDL-C and reduced CVD risk [196,197,198,199]. Large-scale genetic outcome data for CVD risk reduction are still emerging, and the overall population frequency of *IDOL* loss-of-function alleles is relatively low compared to *PCSK9* or *ANGPTL3*.

#### 5.7.2. Preclinical Supporting Evidence

Advancement towards clinical translation of IDOL’s therapeutic potential has been hampered by critical differences between IDOL biology in humans and monkeys when compared to the extensive research in mouse models [200]. Rabbits better approximate human lipid metabolism overall, providing superior models in preclinical settings for CVD research and beyond. Our lab therefore engineered IDOL KO rabbits by employing CRISPR/Cas9 genome editing, resulting in the complete loss of IDOL function and consequently elevated protein levels of its downstream targets. When challenged with a high-cholesterol diet (HCD), IDOL KO rabbits had markedly reduced plasma concentrations of TC and TG compared with WT rabbits. As a result, IDOL deficiency significantly attenuated the development of atherosclerosis [201]. These findings collectively support IDOL inhibition as a promising strategy for the simultaneous reduction in TC, TG, and CVD risk.

#### 5.7.3. Risk Considerations

Gene editing *IDOL* to prevent cardiovascular disease could disrupt cholesterol homeostasis by causing excessive LDLR activity, potentially resulting in abnormal hepatic cholesterol accumulation and liver steatosis or dysfunction. Since IDOL also regulates VLDLR and ApoER2, its loss may affect lipid metabolism in peripheral tissues and interfere with neuronal signaling and brain development, given these receptors’ roles in the central nervous system [202,203].

### 5.8. General Limitations of These Trials

Existing LivGETx-CVD trials (Table 2) mostly focus on a rare indication, i.e., dyslipidemia. Although these trials provide proof of concept for durable LDL-C or triglyceride lowering after editing the listed target genes, they remain early-phase and in small scale. In contrast, CVD, in general, and atherosclerosis, in particular, are chronic and often last for decades and affect millions of patients. Hence, current trials are underpowered to detect infrequent but potentially catastrophic events such as treatment-emergent malignancy, delayed hepatotoxicity, or immune-mediated reactions to editors or delivery vehicles that could arise decades later in life.

Moreover, participants are typically selected for relatively preserved hepatic function, controlled comorbidities, and intensive specialist follow-up, all of which limit the generalizability of these trials to the broader CVD population that commonly includes older adults with other disease conditions such as chronic kidney disease and metabolic syndrome. As such, before LivGETx-CVD can be contemplated for primary prevention or moderate-risk patients, much larger, longer, and more diverse trials will be required, which are trials that not only track traditional cardiovascular endpoints but also rigorously characterize off-target mutational spectra, clonal hematopoiesis, neurocognitive outcomes, reproductive health, and multi-generational effects where feasible.

**Table 3:** Summary of active LivGETx-CVD trials as of 1 November 2025.

Trial	Company	Editor Type	Editing Target	Mutation Type	Delivery Method	Indication(s)	Phase/Status	Main Results/Outcomes (Reference)
VERVE-101	Verve Therapeutics	Adenine base editor	*PCSK9*	Loss-of-function	LNP (IV)	HeFH	Phase 1b	Up to 55% LDL-C↓; durable PCSK9 KO; good safety [204]
VERVE-102	Verve Therapeutics	Adenine base editor	*PCSK9*	Loss-of-function	LNP (IV)	HeFH, premature CAD	Phase 1b	Up to 69% LDL-C↓, 84% PCSK9↓; no treatment SAEs [205]
ART002	AccurEdit Therapeutics	Canonical Cas9 nuclease	*PCSK9*	Loss-of-function	LNP (IV)	HeFH	Single-arm IIT complete	>50% LDL-C↓, good safety; triglyceride data not highlighted [120]
YOLT-101	YolTech Therapeutics	Adenine base editor	*PCSK9*	Loss-of-function	LNP (IV)	HeFH	Phase 1	50% LDL-C↓, >70% PCSK9↓ at 4 months, mild transient events [206]
VERVE-201	Verve Therapeutics	Adenine base editor	*ANGPTL3*	Loss-of-function	GalNAc-LNP (IV)	RH, HoFH	Phase 1b Ongoing	Preclinical: 98–99% ANGPTL3↓; clinical data pending [207]
CTX310	CRISPR Therapeutics	Canonical Cas9 nuclease	*ANGPTL3*	Loss-of-function	LNP (IV)	FH, SHTG, mixed dyslipidemia	Phase 1	Up to 82% TG↓, up to 86% LDL-C↓, well-tolerated [208]
CS121	Correctseq	Transformer base editor	*APOC3*	Loss-of-function	LNP (IV)	FCS, severe hypertriglyceridemia	Phase 1	Rapid TG↓ (initial patient); no adverse events; longer safety pending [209]
CTX320	CRISPR Therapeutics	Canonical Cas9 nuclease	*LPA*	Loss-of-function	LNP (IV)	Elevated Lp(a), ASCVD/aortic stenosis	Phase 1 Ongoing	Preclinical: 95% Lp(a)↓; clinical data awaited [182]

LNP (IV): Lipid nanoparticle, intravenous delivery. HeFH: Heterozygous familial hypercholesterolemia. FH: Familial hypercholesterolemia. RH: Refractory hypercholesterolemia. HoFH: Homozygous familial hypercholesterolemia. SHTG: Severe hypertriglyceridemia. TG↓: triglyceride reduction. LDL-C↓: LDL cholesterol reduction. PCSK9 KO: Knockout of the PCSK9 gene. PCSK9↓: PCSK9 downregulation. ANGPTL3↓: ANGPTL3 downregulation. SAEs: Serious adverse events. All table data are drawn from the most recent primary updates and clinical trial listings as of November 2025.

## 6. Challenges of LivGETx-CVD

### 6.1. Delivery Related Challenges

Delivery is a major challenge for LivGETx-CVD and for GETx in general. Low delivery efficiency may require higher doses, which can increase the risk of adverse events. While several delivery vehicles exist, each comes with its own strengths and weaknesses. To address efficiency, researchers are optimizing carrier systems like LNPs and viral vectors for improved cellular uptake, endosomal escape, and nuclear localization [80,210].

Some delivery vehicles, especially viral vectors, can trigger immune responses, or prolonged exposure may increase the risk of off-target editing. For enhanced safety, strategies have focused on minimizing immune responses [211,212] and limiting sustained exposure to CRISPR/Cas9 components [213,214].

Specificity remains another major limitation for CRISPR/Cas9 delivery. Achieving targeted delivery to the correct tissue or cell type is crucial to maximizing therapeutic benefit and reducing off-target effects, especially when targeting challenging sites like tissues protected by the blood–brain barrier. Specificity is improved by adding targeting ligands, antibodies, or aptamers that guide the editing machinery to particular tissues or cell types [79], as well as utilizing tissue-specific or inducible promoters to control Cas9 expression only in desired cells.

We want to point out that despite the challenges associated with delivery methods, including issues of efficiency, tropism, and safety, one consistent outcome is that the liver tends to be the predominant site of cargo deposition. Rather than being viewed solely as a limitation, this natural bias toward hepatic targeting can be leveraged as a therapeutic advantage, particularly for GETx aimed at treating dyslipidemia, since many of the key genes that are involved in lipid metabolism and regulation are primarily expressed in the liver.

### 6.2. Off-Target Effect

Off-target editing refers to the unintended cleavage and modification of genomic sequences at sites other than the intended site by CRISPR/Cas9 genome editing [215]. This phenomenon can introduce unwanted mutations in other regions of the genome, potentially disrupting normal gene function or even leading to oncogenic effects. This is an inherent problem of gene editing nucleases, hence applicable to LivGETx-CVD as well.

A primary approach to address this concern involves engineering Cas9 proteins to increase their specificity. High-fidelity Cas9 variants, such as Cas9-HF1 [216], enhanced specificity Cas9 (eSpCas9) [217], HiFi-Cas9 [218] and evoCas9 [219] are modified at specific amino acid positions to reduce non-specific DNA interactions, thereby minimizing off-target cleavage. Cas9 nickase can also reduce off-target editing because two adjacent single-strand nicks are required, each guided by its own gRNA, to generate a DSB at the intended site. This means that successful editing occurs only when two guides bind correctly and close together at the target sequence, which is much less likely to occur at unintended sites [220].

Another effective method is to optimize gRNA design. By truncating the length [221] or introducing chemical modifications into gRNAs [222], scientists can improve their specificity. Computational tools are also widely used for selecting and validating gRNA sequences with minimal similarity to non-target genomic loci [223].

Finally, reducing the duration of Cas9 activity in cells is crucial. Persistent expression of Cas9 increases the likelihood of prolonged, unwanted activity. One of the most effective approaches is the delivery of an RNP complex, which is quickly degraded after performing genome editing. Alternatively, using non-integrating delivery vehicles, such as adenoviral vectors, LNPs, or eVLPs, can also provide transient expression without permanent integration into the genome. Additionally, using switches to regulate Cas9 expression allows researchers to turn Cas9 activity on or off in response to specific stimuli, thereby enhancing genome editing precision and safety. Common types include small molecule-inducible systems (e.g., doxycycline-controlled Tet-On/Tet-Off or rapamycin) [213] and light-activated switches [214].

### 6.3. On-Target Genotoxicity

On-target undesired editing occurs when the CRISPR/Cas9 system introduces unwanted genetic changes at the intended genomic site. In GETx applications where DSB-capable Cas9 is used, a double-strand break (DSB) is produced on-target. HDR is much less efficient than NHEJ in most cells, so the majority of repairs lead to small errors. Additionally, the repair process can result in larger structural variants (SVs) such as large deletions, insertions, inversions, translocations, or even integrations of foreign DNA [224,225].

To favor precise editing, researchers have developed several strategies to promote HDR over NHEJ: using small-molecule enhancers to stimulate HDR pathway proteins such as RAD51 or CtIP [226,227]; using inhibitors of NHEJ pathway proteins such as Ku70-80, DNA ligase IV, or DNA-PKcs [228,229,230]; arresting cells in S or G2 phases when HDR is most active [231]; and supplying donor DNA in ssODNs format that increases availability [232]. Additionally, engineering Cas9 proteins fused to motifs or proteins that recruit HDR pathway proteins, such as RAD51 or CtIP, to the cut site can directly stimulate HDR [222,223].

An alternative solution is to use genome editing tools that do not create DSBs at all. For example, base editors allow targeted point mutations without double-strand breaks. Prime editing offers more flexibility, making insertions, deletions, and base-to-base conversions while avoiding the risks associated with DSBs. However, both BE and PE systems can still cause substantial undesirable on-target edits. Base editors often result in bystander edits, where nucleotide changes occur at unintended positions within the editable window, potentially altering nearby bases unintentionally. In addition, both base editors and prime editors can generate insertion and deletion (indel) mutations at the target site, which may disrupt gene function or cause genomic instability. These limitations highlight the need for improved specificity and control in next-generation gene editing technologies.

### 6.4. Immunogenicity

Immunogenicity is a major consideration for the clinical application of CRISPR/Cas9-based therapies, as the introduction of foreign gene editing components can provoke immune responses that compromise safety and efficacy. Again, this is a general risk for all GETx, hence also applicable to LivGETx-CVD.

The most commonly used Cas9 protein is derived from *Streptococcus pyogenes*, a bacterium well known to the human immune system due to its role in many common infections [224]. As a result, the host immune system may rapidly recognize Cas9 as a foreign antigen, triggering immune responses that could eliminate the protein before gene editing is completed. To address this, researchers have engineered the Cas9 protein with reduced immunogenicity by deleting or altering immunogenic epitopes [211] and have also explored using Cas9 orthologs from less prevalent bacteria to reduce the risk of pre-existing immunity.

In addition, gRNAs themselves can induce innate immune responses. This risk can be mitigated by phosphatase treatment that reduces RNA immunogenicity by removing the 5′ triphosphate groups, which are potent activators of the innate immune system [212].

For further safety, gene editing therapies can be used to target immune-privileged sites in the body, such as the eyes, brain, fetus, or testes, where local immune response to foreign antigens is naturally limited, lowering the likelihood of an adverse immune reaction and increasing the chance of successful editing.

### 6.5. Further Considerations

Although it is not the primary mission of a research review article, we wish to highlight additional considerations that must be addressed on the path to implementing LivGETx in the clinic. These represent a distinct set of challenges that go beyond technical optimization of editing tools and delivery systems and require sustained dialog among clinicians, regulators, ethicists, economists, and the broader public.

#### 6.5.1. From Rare Disease to Prevention

At present, LivGETx-CVD is tested in highly selected individuals with severe dyslipidemia, limited alternatives, and very high event rates, where one-time disruption of targets such as PCSK9, ANGPTL3, APOC3, or LPA can be ethically justified by substantial potential benefit.

Extending this paradigm to the far larger population with common multifactorial cardiovascular risk would shift LivGETx from treating rare disorders to preventing disease in otherwise healthy people, requiring societal consensus on risk thresholds that justify permanent genomic modification, acceptable pricing, and how such interventions should coexist with reversible therapies, among others.

#### 6.5.2. Cost-Effectiveness and Feasibility

The “one-shot, one-cure” vision positions LivGETx-CVD as analogous to vaccination, potentially offering durable lipid-lowering for patients who cannot tolerate, access, or adhere to chronic lipid-lowering regimens.

However, the complexity of vector or delivery vehicle (e.g., LNP) manufacturing, individualized dosing, specialized infusion centers, intensive early monitoring, and prolonged follow-up will initially drive high per-patient costs, so health-economic analyses must weigh durability and infrastructure demands against inexpensive oral agents (e.g., Statins) and injectables (e.g., PCSK9 mAbs), while considering that standardized manufacturing and price reductions could eventually make a vaccine-like strategy attractive in settings with fragile supply chains and poor long-term adherence.

#### 6.5.3. Ethical and Societal Questions

At the societal level, in vivo editing of lipid-related genes for cardiovascular prevention blurs the boundary between treatment and enhancement, especially when edits are introduced early in life or in individuals who are currently healthy but have elevated long-term risk. Societal unease is likely to intensify if editing is used not only to neutralize high-risk alleles (for example, PCSK9 gain-of-function) but to introduce “superior” alleles that confer unusually low LDL-C or triglyceride levels, raising questions about fairness, pressure to undergo enhancement, and what constitutes an acceptable level of cardiovascular risk in otherwise healthy individuals.

Parallel worries surround distributive justice: if single-dose editing is costly and concentrated in well-resourced health systems, it could become a “rich people’s game” that amplifies existing disparities in cardiovascular outcomes between and within countries.

At the individual level, in addition to “body-level” outcomes, psychological and behavioral effects deserve systematic study: recipients may experience persistent anxiety about irreversible genomic alteration, altered self-identity, or risk-compensation behaviors (for example, reduced adherence to diet and exercise) once they perceive themselves as “vaccinated” against cardiovascular disease. Robust registries and post-marketing surveillance frameworks analogous to those for gene transfer and cell therapies will be essential to capture these dimensions across diverse populations.

Another central ethical concern at the individual level, which extends to the societal level, is the potential germline transmission, i.e., if editing components reach gonadal tissue and edited alleles are transmitted to the next generation through germ cells. This is a risk that preclinical studies monitor carefully but cannot yet exclude with absolute certainty. Adding another layer of complexity to this concern is whether or not such an edited allele should be allowed to be introduced to the human gene pool. These issues argue for early engagement of patient communities and the public in setting acceptable indications, age windows, and risk thresholds for LivGETx-CVD (which, in fact, is applicable to all gene editing therapies), rather than leaving decisions solely to developers and payers.

## 7. Conclusions

LivGETx-CVD, i.e., gene editing-based therapy targeting the liver, holds promise as a “one-shot” treatment for dyslipidemia, a condition that has traditionally required lifelong management, and for cardiovascular diseases in general. Achieving this potential will require sustained investment in the development of advanced gene editing tools, the identification of novel therapeutic targets, the optimization of delivery systems, continued efforts to address current challenges related to efficacy and safety, as well as reaching a societal consensus on ethical and regulatory issues.

## Figures and Tables

**Table 1:** Primary features of major potential delivery systems to achieve LivGETx-CVD.

Platform (Reference)	Cargo Type	Cargo Size	Immunogenicity	Genome Integration	Tissue Tropism	Particle Size
AAV [39]	DNA	~4.7 kb	Low-moderate	Rare (episomal)	Broad, serotype-dependent	~20–25 nm
AdV [40]	DNA	~8–36 kb	High	No	Broad (liver, airway, etc.)	~70–100 nm
LV [41]	RNA (retroviral)	~8–10 kb	Moderate	Yes (integrates)	Broad, envelope-dependent	~80–120 nm
VLP [42]	Protein or peptide	Variable (<150 kDa protein typical)	Low	No	Customizable by engineering	~20–200 nm (system-dependent)
LNP [43]	DNA, mRNA, protein, or small molecules	Up to ~15 kb mRNA	Low-moderate	No	Liver primarily, or engineered targeting	~60–150 nm

**Table 2:** Summary of loss-of-function effects of targeted genes.

Gene	Key Mechanism Exploited in LivGETx-CVD	Hypothesized Loss of Function Effects in LivGETx-CVD
*PCSK9*	Degrade LDLR	Retention of LDLR → reduced cholesterol in the blood → less CVD risks
*ANGPTL3*	Inhibit LPL	Less inhibition of LPL → increased digestion of triglycerides → reduced triglyceride levels in the blood → less CVD risks
*CETP*	Convert HDL to LDL	Less conversion of HDL to LDL → increased HDL-C and decreased LDL-C levels in the blood → less CVD risks
*ApoC3*	Inhibit LPL	Less inhibition of LPL → increased digestion of triglycerides → reduced triglyceride levels in the blood → less CVD risks
*ASGR1*	Inhibition of ASGR1 promotes bile excretion	Upregulation of reverse cholesterol transport → increased cholesterol excretion through feces → reduced cholesterol levels → less CVD risks
*LPA*	Pro-inflammation	Less inflammation → less CVD risks
*IDOL*	Degrade LDLR	Retention of LDLR → reduced cholesterol → less CVD risks

## Data Availability

No new data were created or analyzed in this study.
